# Engineering of *Salmonella* Phages into Novel Antimicrobial Tailocins

**DOI:** 10.3390/cells12222637

**Published:** 2023-11-16

**Authors:** Cedric Woudstra, Anders Nørgaard Sørensen, Lone Brøndsted

**Affiliations:** Department of Veterinary and Animal Sciences, University of Copenhagen, 1870 Frederiksberg C, Denmark; cedric.woudstra@sund.ku.dk (C.W.); anders.norgaard@sund.ku.dk (A.N.S.)

**Keywords:** Tailocin, head-less phage, bacteriocin, genetic engineering, *Ackermannviridae*

## Abstract

Due to the extensive use of antibiotics, the increase of infections caused by antibiotic-resistant bacteria is now a global health concern. Phages have proven useful for treating bacterial infections and represent a promising alternative or complement to antibiotic treatment. Yet, other alternatives exist, such as bacteria-produced non-replicative protein complexes that can kill their targeted bacteria by puncturing their membrane (Tailocins). To expand the repertoire of Tailocins available, we suggest a new approach that transforms phages into Tailocins. Here, we genetically engineered the virulent *Ackermannviridae* phage S117, as well as temperate phages Fels-1, -2 and Gifsy-1 and -2, targeting the food pathogen *Salmonella*, by deleting the *portal vertex* or *major capsid* gene using CRISPR-Cas9. We report the production of Tailocin particles from engineered virulent and temperate phages able to kill their native host. Our work represents a steppingstone that taps into the huge diversity of phages and transforms them into versatile puncturing new antimicrobials.

## 1. Introduction

Bacteriophages (phages) are natural predators of bacteria that are concomitantly present wherever a suitable host is found. Phages have been used for more than one century to treat bacterial infections, mostly in Eastern countries [1]. The Eliava Institute in Georgia, for example [2], has been providing phage preparations to treat bacterial infections of *Staphylococcus*, *Streptococcus*, *Pseudomonas aeruginosa*, *Proteus*, *E. coli*, *Salmonella*, and others since 1923 [3]. Today, phages are recognized as a possible alternative and/or complement to the classical treatment (e.g., antibiotics) of bacterial infections and are successfully used to treat resistant bacterial infection [4,5]. Due to the increase of acquired resistance to available antibiotic treatment, multidrug-resistant bacteria are currently responsible for about 15.5% of hospital-acquired infection cases [6]. One of the most spectacular and mediatic examples is probably the story of Tom Patterson, who suffered an infection with *Acinetobacter baumannii* and recovered miraculously after being injected with a personalized phage treatment [7]. 

While phages are promising antimicrobial agents for targeting antibiotic-resistant bacteria, other strategies using phage-related particles can be devised. Tailocins, also called Bacteriocin phage-like particles, are natural non-replicative high-molecular-mass protein complexes that resemble phage tails with *Myoviridae* or *Siphoviridae* morphologies [8]. Tailocins consist of a contractile (R-type) or non-contractile (F-type) sheath that forms a rigid tube attached to a baseplate with a spike-shaped protein complex at its tip and associated receptor binding protein (RBP) [9]. They kill by perforating the bacteria cell wall and leading to membrane potential collapse [10]. Tailocins have obtained increasing interest in the last decade for their potential use as therapeutics to fight antibiotic-resistant bacteria [11,12]. For example, a recent study characterized a Tailocin with a broad host range against *Burkholderia* spp., which is known to acquire antibiotic resistance and represents a significant threat to cystic fibrosis patients [13]. Tailocins have also been used as biocontrol against phytopathogenic bacteria [14]. For example, the Tailocin produced by *Pseudomonas fluorescens* SF4c was successfully used to control the bacterial-spot disease in tomatoes caused by *Xanthomonas vesicatoria* [15]. Interestingly, Tailocins can be genetically engineered to change their host range by altering their RBPs. The RBP from the R2 Tailocin produced by *Pseudomonas aeruginosa* was replaced by the RBP from phage PS17, resulting in the death of a different subset of *Pseudomonas aeruginosa* strains than the native Tailocin [16]. These few examples illustrate how Tailocins can be appealing as new antimicrobials. Moreover, Tailocins can theoretically kill one target cell per particle (R-type [10]), do not carry genetic material (and therefore do not transduce DNA), and are non-replicative and, therefore, could be easier to validate administratively as protein complexes for therapeutic use [17]. Yet, while Tailocins have been isolated in different bacteria genera, only a limited number are currently characterized [18]. 

To harness the therapeutic potential of Tailocins against bacteria causing human infections, we suggest a new approach that engineers both virulent and temperate phages into versatile Tailocins by preventing head-to-tail connection. Considering the huge diversity of phages, transforming virulent or temperate phages into Tailocins may provide a large source of antimicrobials for targeting diverse pathogenic bacteria. In phage T4, the portal protein constituting the initiator complex of a 12-mer ring and the major capsid protein that polymerizes on the portal complex are required for correct head formation and head-to-tail assembly [19,20,21]. Thus, the *portal* and *major capsid* genes constitute suitable targets to generate Tailocins from phages. As our bacterial target, we selected the food pathogen *Salmonella enterica* Typhimurium (*S.* Typhimurium), since clones showing antibiotic resistance are increasingly isolated [22]. Among phages infecting *S.* Typhimurium, *Ackermannviridae* are attractive to develop into Tailocins, as they encode up to five tail spike proteins (TSPs) [23]. Each individual TSP functions as an RBP by recognizing specific O-antigen or K-antigen receptors from different bacterial hosts, such as *Salmonella*, *Shigella, Enterobacter, Dickeya, Klebsiella*, and *Serratia* [24]. Thus, the transformation of the broad host range of *Ackermannviridae* phages into Tailocins may be used to fight O- and K-antigen expressing bacteria, such as *Salmonella* and *Klebsiella* spp. [25]. Temperate phages Fels-1, -2, and Gifsy-1 and -2 are widespread *Salmonella* temperate phages that show *Siphoviridae* and *Myoviridae* morphologies. While temperate phages Gifsy-1 and -2 bind to OmpC as receptors [26], Fels-1 and -2 receptors have not been experimentally determined but may be different from OmpC. Thus, the genetic engineering of these temperate phages into Tailocins may transform *Salmonella* into a Tailocin factory that produces a cocktail of contractile and non-contractile Tailocin particles targeting the same host but potentially different receptors. 

In this study, we aimed to increase the versatility of phages by transforming them into Tailocins. *Ackermannviridae* virulent phage S117 as well as *S.* Typhimurium temperate phages Fels-1, -2 and Gifsy-1, -2 were genetically engineered using CRISPR-Cas9 to be deficient for the *portal* or the *major capsid* gene. Their deletion allowed for the production of Tailocin particles derived from phage S117, as well as Fels-1, -2 and Gifsy-1, -2 Tailocin particles that were characterized for their ability to kill *S.* Typhimurium. 

## 2. Materials and Methods

### 2.1. Bacteriophage, Bacterial Strain, and Culturing Media

*S.* Typhimurium LT2C, devoid of prophages [27], was used as the host for engineering the *Ackermannviridae* virulent phage S117 [24]. *S.* Typhimurium 3674 was used as the native host for engineering temperate phages Fels-1, -2 and Gifsy-1, -2 [28]. Luria agar 1.5% (LA), Luria agar 0.6%, and Luria broth (LB) were used to cultivate *S.* Typhimurium on solid or in liquid media, with or without antibiotics (kanamycin 50 μg/mL, spectinomycin 50 μg/mL). 

### 2.2. From Phage DNA to Cloned Plasmids

Phage DNA was extracted using a rapid DNA extraction method [29]. Briefly, a phage stock solution at 10^11^ pfu/mL was incubated with 50 µL of DNase I 10x buffer, 1 µL of DNase I (1 U/µL), and 1 µL of RNase A (10 mg/mL) for 1.5 h at 37 °C without shaking. Following this, 20 µL of 0.5 M EDTA (final concentration 20 mM) was added to inactivate DNase I and RNase A. The phage protein capsid was digested using 1.25 µL of Proteinase K (20 mg/mL) and was incubated for 1.5 h at 56 °C without shaking. DNA amplification of phage or bacterial genetic sequences was performed by PCR using the CloneAmp HiFi PCR premix^®^ (Takara Bio©, Saint-Germain-en-Laye, France). Primers used in this study are listed in Table S1. PCR amplicons were purified using the Zymo PCR purification kit^®^ (Zymo Research©, Freiburg im Breisgau, Germany). Plasmid constructions were performed using the In-fusion^®^ HD-cloning kit (Takara Bio©, Saint-Germain-en-Laye, France) in highly competent Stellar *E. coli* cells. Constructed plasmids were extracted using the GeneJET Plasmid Miniprep Kit^®^ (Thermo Fisher Scientific©, Roskilde, Denmark). All procedures followed the manufacturer’s instructions. DNA concentration was measured with Qubit (Thermo Fisher Scientific©, Roskilde, Denmark), and DNA quality was verified on a 1% agarose gel. Plasmids were sequence-verified by Sanger sequencing (Eurofins Genomics©, Ebersberg, Germany).

### 2.3. Genetic Engineering

A double CRISPR-Cas plasmid system (pEcCas, pEcgRNA [30]) was used to engineer phage S117, while phages Fels-1, -2 and Gifsy-1, -2 were engineered using homologous recombination linear DNA fragments. Plasmid pEcCas contains kanamycin resistance (Addgene Plasmid #73227), and plasmid pEcgRNA (Addgene Plasmid #166581) contains spectinomycin resistance. The plasmid pEcCas encodes Cas9 under a constitutive promoter, as well as the Lambda-Red system under an arabinose inducible promoter. The plasmid pEcgRNA contains the guide that targets Cas9 to a specific genetic region and the homologous recombination DNA sequence used to delete the gene of interest. Importantly, strain *S.* Typhimurium LT2C showed to be not transformable with plasmid pEcCas, due to the presence of multiple restriction-modification (RM) systems. We overcame this problem by cloning the type III RM methyltransferase StyLTI (STM0357 from NC_003197.2) in *E. coli* and transformed pEcCas in our StyLTI _m+ *E. coli*. We could then extract the pEcCas methylated in *E. coli* and transform it back in *S.* Typhimurium LT2C. 

### 2.4. CRISPR-Cas gRNA Efficiency against S117

Each guide was evaluated for its efficiency against the phage S117. The *S.* Typhimurium LT2C strain was transformed with pEcCas and pEcgRNA-g1 to pEcgRNA-g20. The protection provided by each guide was measured individually by comparing the reduction of infection by a double-layer plaque assay, when infected by serial dilution of phage S117. Briefly, the LT2C strain containing pEcCas and pEcgRNA-g was grown exponentially to OD600 0.5. A total of 100 µL of LT2C was mixed with 4 mL of top agar 0.6% and poured onto an LA plate containing kanamycin (50 mg/mL) and spectinomycin (50 mg/mL) to select for bacteria containing both pEcCas and pEcgRNA. Serial dilutions of phage stock S117 were then spotted (10 µL) onto the surface of the dried plate. The plate was incubated at 37 °C overnight, and plaques were counted the next day and compared to a negative control of LT2C containing only the pEcCas plasmid spotted with the same serial dilution of S117. The guide showing the highest reduction in S117 infectivity was selected for further downstream application. 

### 2.5. Construction of pEcgRNA Plasmid to Engineer S117

Primers used to construct pEcgRNA are available in Table S1. Plasmid pEcgRNA used to genetically engineer phage S117 was built in three steps. First, pEcgRNA was reverse amplified by PCR to include the most successful sgRNA guide under the constitutive promoter J23119, producing plasmid pEcgRNA-g. Second, plasmid pEcgRNA-g was reverse amplified by PCR and cloned together with the DNA homologous recombination (HR) template to allow for the knockout of the gene of interest, the *portal vertex* of S117, producing plasmid pEcgRNA-g-HR. The HR fragment contains two regions of 500 bp, the left homology arm (LHA) and the right homology arm (RHA) that flank the region to modify (here, the *portal* gene). LHA and RHA were amplified separately from phage S117 DNA and joined together by SOE PCR to form the HR recombination template [30]. Third, pEcgRNA-g-HR was reverse amplified and cloned together with the complementation *portal* gene (*cportal*), producing the plasmid pEcgRNA-g-HR-c*portal* (Figure S1). The *cportal* gene was codon optimized for *S. enterica* to avoid pEcgRNA-g-HR-cportal being self-targeted. The *cportal* was ordered commercially (IDT^®^, Leuven, Belgium). The complementation *cportal* had 78% nucleotide similarity and 100% amino acid identity to the phage S117 native portal protein. This allowed for the production of fully functional phage particles containing the S117 genome deleted for the *portal* gene. 

### 2.6. S117 Portal Gene Deletion

The LT2C strain containing pEcCas and pEcgRNA-g-HR-*cportal* was grown at 37˚C until the exponential phase at OD_600 nm_ 0.5, together with arabinose (0.1%), to induce the Lambda-Red system on pEcCas. Then, 100 µL of culture was mixed with 4 mL of LA 0.6% and poured onto an LA plate containing kanamycin (50 μg/mL) and spectinomycin (50 μg/mL). After drying for 10 min, 10 µL of the serial dilution of phage S117 stock (10^10^ pfu/mL) was spotted onto the surface and dried for 15 min. After overnight incubation, single plaques visible at the highest dilution were picked with a toothpick and resuspended into SM phage buffer [31] and tested by PCR for the deletion of the *portal* gene. Plaques that were positive for the *portal* deletion were used in a new round of infection. The process was repeated three times to ensure the purity of the recombinant S117Δ*portal* deleted for the *portal* gene.

### 2.7. Fels-1, -2, Gifsy-1, -2 Major Capsid Gene Deletion

The *S.* Typhimurium strain 3674 containing pEcCas was grown at 37 °C to the exponential phase at OD_600 nm_ 0.5, together with arabinose (0.1%), to induce the Lambda-Red system on pEcCas for homologous recombination. The cells were then harvested and washed 3 times with water. The cells were electroporated (MicroPulser Electroporator Bio Rad with settings: Ec1 (V = 1.8 kV) for 0.1 cm cuvettes and Ec2 (V = 2.5 kV) for 0.2 cm cuvettes) with 250 ng of linear PCR product of a homologous recombination fragment targeting Fels-1 *major capsid protein* gene (*MCP*) to replace it with a chloramphenicol cassette CmR (Figure S2). The electroporated cells were resuspended into 500 µL of warmed LB media and incubated for 1 h at 37 °C under shaking at 180 rpm. Amounts of 100 µL, 10 µL, and 1 µL were then spread onto LA plates containing kanamycin and chloramphenicol at 50 μg/mL and incubated overnight at 37 °C. Single colonies were then tested by PCR to check for deletion. A single colony containing pEcCas that was deleted for Fels-1 *major capsid* gene was chosen. The clone empty of Fels-1 *MCP* was then used, and the procedure was repeated with a new homologous recombination PCR product targeting the *MCP* of Fels-2, then Gifsy-1 and then Gifsy-2, with a different antibiotic cassette (Figures S3–S5), until it produced a strain engineered to be devoid of all *major capsid* genes from Fels-1, -2, Gifsy-1, and Gifsy-2.

### 2.8. Tailocin Production: S117 Tailocin

One liter of exponentially grown LT2C was infected with S117Δ*portal* at MOI (multiplicity of infection) 0.1, 1, 10, and 100. Samples of 35 mL were taken after 10, 20, 30, 40, and 50 min of infection to evaluate the phage adsorption efficiency and the optimal time for Tailocin particle release. Samples were then centrifuged at 8000 rpm for 10 min at 4 °C. The supernatant was then filtered twice with a 0.45 µm and 0.22 µm filter and kept at 4 °C for further use. The presence of phage and Tailocin particles was assessed by spot assay on *S.* Typhimurium LT2C. Tailocin particles were further concentrated by PEG precipitation. A solution of PEG_8000_ at 30% in a solution of 1.5 M NaCl (autoclaved) was used to precipitate the Tailocin particles by adding 1/3 of the PEG solution to 2/3 of the Tailocin particles. After 48 h at 4 °C, the suspension was centrifuged at 12,000 rpm for 2 h, and the supernatant was discarded. The pellet was then resuspended in 500 µL of SM phage buffer. Tailocin particles were also separately concentrated using 50 KDa Amicon^®^ purification columns (Millipore©, Søborg, Denmark).

### 2.9. Tailocin Production: Fels-1, -2, Gifsy-1, -2 Tailocins

One liter of exponentially grown strain 3674 deleted for the *major capsid* genes of Fels-1, -2 and Gifsy-1, -2 was submitted to mitomycin C treatment at 2 µg/mL for 3 h. The cells were then centrifuged for 20 min at 8000 rpm, and the supernatant was sterilized using 0.45 µm and then 0.22 µm filters. The supernatant was kept at 4 °C for two weeks prior to being used to allow for the degradation of mitomycin C. Further concentration was also performed using PEG_8000_ at 30%, as previously described in Section 2.8.

### 2.10. Tailocin Killing Assay

A total of 100 µL of exponentially grown *S.* Typhimurium strain was mixed with 4 mL of LA 0.6% and poured onto an LA plate. The Tailocin solutions were then tested by spotting 10 µL of serial dilutions onto the bacterial lawn. A result was considered positive when a lysis ring was observed at the place of the drop. 

## 3. Results

### 3.1. Overall Approach for Engineering of Phage S117 into a Tailocin

**Figure 1:**
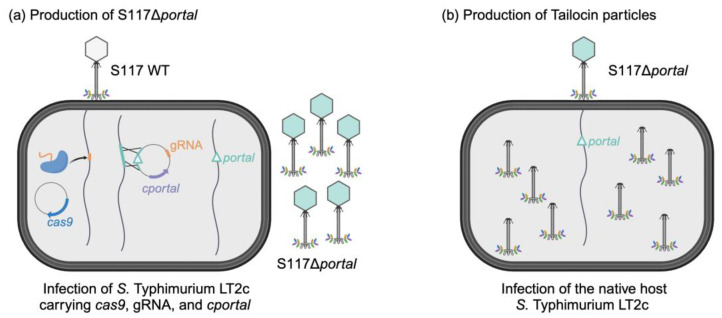
Engineering strategy to produce Tailocin particles from phage S117. (**a**) The production of phage S117Δ*portal*. A native phage S117 infects *S.* Typhimurium LT2C with the plasmid pEcCas containing *cas9* and pEcgRNA. A guide RNA on the pEcgRNA plasmid allowed Cas9 to target and cut the wild-type phage genome at the *portal* gene. pEcgRNA contains homologous recombination sequences that allowed the phage genome to escape Cas9 by deleting the *portal* gene. In the presence of the complementation *cportal*, the portal protein was produced constitutively and allowed production of phage particles containing the phage genome deleted for the *portal* gene. (**b**) The production of Tailocin particles from phage S117Δ*portal.* The engineered phage S117Δ*portal* was used to infect the native *S*. Typhimurium LT2C host. In the absence of any *portal* gene to complement the engineered phage genome, the portal protein was not synthesized, and only Tailocin particles were produced.

We devised a two-step approach to produce Tailocin from phage S117. First, we engineered the genome of phage S117 by deleting the *portal* gene (S117Δ*portal*) using CRISPR-Cas9 during the infection of *S.* Typhimurium LT2C carrying a complementation *portal* gene *in trans* (Figure 1a). This produced S117Δ*portal* phage particles that lack the *portal* on the genome but phenotypically behave as wild type phages. Second, we used the phage stock of S117Δ*portal* to infect the native host, *S.* Typhimurium LT2C, leading to the production of Tailocin particles (Figure 1b). Thus, to engineer the genome of phage S117, we used a CRISPR-Cas9 approach already proven to efficiently produce phage mutants [32].

### 3.2. Engineering of Phage S117 Lacking the Portal Gene

#### 3.2.1. CRISPR-Cas9 Guides Efficiencies

A key aspect of using CRISPR is to provide the right guide for Cas9 to reach its target [33]. Especially, modified phage DNA makes it difficult to predict guide efficiency [33]. Phage FEC14 and CBA120, which also belong to the *Ackermannviridae* family, have already been shown to be resistant to enzymatic restriction [34,35] due to DNA modification from a deoxyuridylate hydro-methyltransferase. The phage S117 genome (GenBank accession number MH370370) also contains a locus (*orf51* to *orf55*) predicted to be involved in the production of non-canonical nucleotides by substituting thymine by hydroxy-methyluracil. Therefore, phage S117 is expected to show variability in the efficiency of different CRISPR-guides. Consequently, we evaluated 20 different guides targeting the *portal* gene of phage S117 for their efficiency to allow Cas9 to restrict the S117 genome in the *portal* gene. The *portal* gene of phage S117 (*orf149*) is located within the locus of the head and tail assembly, and guides were selected and manually distributed over the entire 1683 bp gene and further checked bioinformatically using CrisprScan [36]. The efficiency of each guide was calculated based on the reduction in the efficiency of plating (EOP, [37]). Phage S117 plaque formation was assessed on a lawn of *S.* Typhimurium LT2C containing pEcCas9 and the guide to the *portal* from phage S117. The results were compared with the number of plaques formed on a lawn of LT2C without pEcCas9 (Table 1). 

As expected, we found that the guides’ efficiency to reduce the EOP of S117 was variable and did not correlate with software prediction, as previously reported for phage T4 [33]. EOP reduction varied from no reduction up to five log reduction in S117 efficiency to infect LT2C. Based on the results, guide 11, showing a five-log reduction of EOP, was selected as more efficient to target S117 *portal* gene.

#### 3.2.2. Production of Engineered S117Δ*portal*

**Figure 2:**
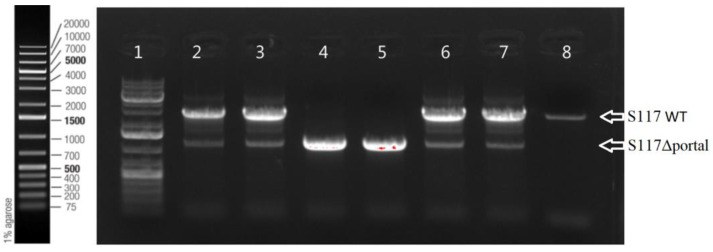
Plaque PCR of the S117Δ*portal* to confirm the absence of the *portal* gene. The presence of the wild-type *portal* gene resulted in a band of 3688 bp (well 8, genomic DNA of S117), but if deleted, the amplification produced a band of 1191 bp. Plaques from well 2, 3, 6, and 7 contained a mix of engineered and native phages. Plaques from wells 4 and 5 contained engineered S117Δ*portal* only. Ladder of 1 Kb in well 1.

To produce phage S117 lacking the *portal* gene, we used wild-type phage S117 to infect *S.* Typhimurium LT2C containing Cas9 (plasmid pEcCas), guide 11, the homologous recombination (HR) fragments LHA and RHA fragments surrounding the *portal* gene, and the complementation c*portal* gene *in trans* (cloned onto pECgRNA, Figure S1). LHA and RHA were selected to be 500 bp long, surrounding the *portal* gene to allow for the genetic recombination exchange to delete the *portal* gene. Single plaques were selected to test for the presence of the genetic recombination. PCR confirmed that the plaques corresponded to the engineered S117Δ*portal* (Figure 2). Some plaques contained a mix of engineered S117Δ*portal* and native S117 (Figure 2). From the plaques containing S117Δ*portal*, three successive rounds of plaque purification and sequencing were performed to ensure that only engineered phages S117 lacking the *portal* were isolated. In summary, after the selection of the best CRISPR-Cas9 guide, it was possible to genetically engineer S117 to lack the *portal* gene.

### 3.3. Characterization of the Engineered Phages S117*Δ*portal

#### 3.3.1. S117Δ*portal* Replication Characterization

**Figure 3:**
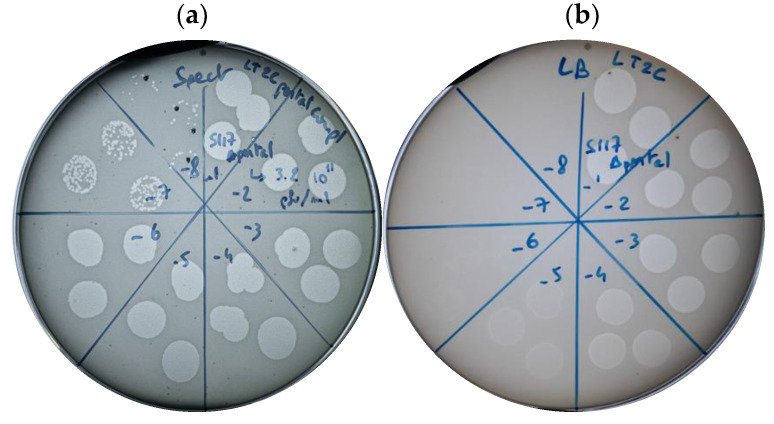
Biological confirmation of engineered S117 deleted for the *portal* gene. (**a**) Phage S117Δ*portal* infection on a lawn of *S*. Typhimurium LT2C containing the complementation *portal* gene. Single plaques were produced up to dilution 10^−7^ and 10^−8^. (**b**) Phage S117Δ*portal* infection on a lawn of *S*. Typhimurium LT2C without the complementation *portal* gene. No single plaques were visible.

To characterize the engineered phage, S117Δ*portal* was propagated on *S.* Typhimurium LT2C containing the *cportal* complementation *in trans* (pECgRNA, Figure S1), producing an S117Δ*portal* phage stock of 3.2 × 10^11^ pfu/mL when plaquing on this strain (Figure 3a). In contrast, the S117Δ*portal* could not form single plaques in the absence of the complemented *cportal* and only formed lysis zones up to 3 × 10^6^ pfu/mL on lawns of *S.* Typhimurium LT2C (Figure 3b). This confirmed that S117Δ*portal* was biologically incapable of producing functional phage particles in the absence of the *cportal* provided *in trans.*

#### 3.3.2. Production of S117Δ*portal* Particles

**Figure 4:**
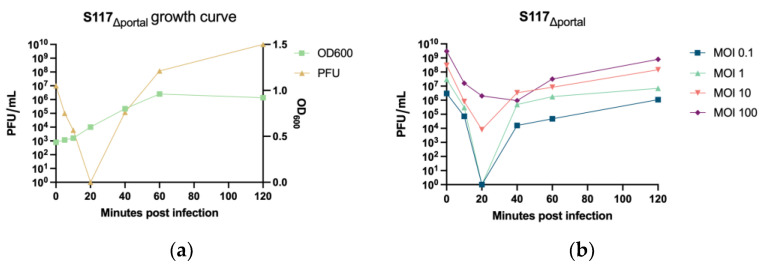
Determination of S117Δ*portal* phage particles released at different MOI. (**a**) Phage particles released at an MOI of 1. On the x axis, the time points are shown in minutes of the host culture. On the y axis on the left, the amount of phage calculated is shown as plaque forming unit (pfu) per mL. On the y axis on the right, the OD_600_ values of *S*. Typhimurium LT2C are shown carrying the complementation *portal* during infection. (**b**) Phage particles released at different MOI. On the x axis, the time points are shown in minutes of the host culture. On the y axis on the left, the amount of phage is shown calculated as plaque forming unit (pfu).

We further investigated the production of phage particles from S117Δ*portal*. *S*. Typhimurium LT2C carrying the complementation *cportal* was infected by phage S117Δ*portal* at an MOI of 1. The production of phage particles as well as the effect on the host population were monitored (Figure 4a) by measuring phage free particles in the supernatant and the remaining host cells after spinning down. 

At MOI 1, 100% of the phage particles of S117Δ*portal* absorbed onto their host within 20 min. The first release of new phage particles was observed at 40 min post-infection and resulted in 10^6^ phage particles per mL. These results are concordant with the previous study of kuttervirus phage CBA120, also belonging to the *Ackermannviridae* family [34]. The experiment was repeated with 0.1, 10, and 100 MOI to determine the influence of the MOI on the infection kinetics (Figure 4b). At an MOI of 0.1, all phage particles were adsorbed after 20 min post-infection, whereas at MOIs 10 and 100, not all phage particles were adsorbed at 20 min post-infection. After 40 min post-infection, the amount of phage particles present was 10^4^, 10^6^, and 10^7^ pfu/mL for MOI 0.1, 10, and 100, respectively. Importantly, at MOI 10 and 100, the total amount of phage particles detected also included those that did not bind immediately to the host, whereas the phage particles found at MOI 0.1 and 1 only included those produced, as there were no remaining free phage particles after 20 min post-infection. Therefore, a lower MOI allowed for a higher production of S117 phage particles at early post-infection time points. In summary, we successfully constructed phage S117 lacking the *portal* gene, which, when complemented by the *portal* protein *in trans*, showed a similar growth pattern to other *Kuttervirus* phages.

### 3.4. Tailocin Particles Produced from Phage S117*Δ*portal Form Inhibition Zones

**Figure 5:**
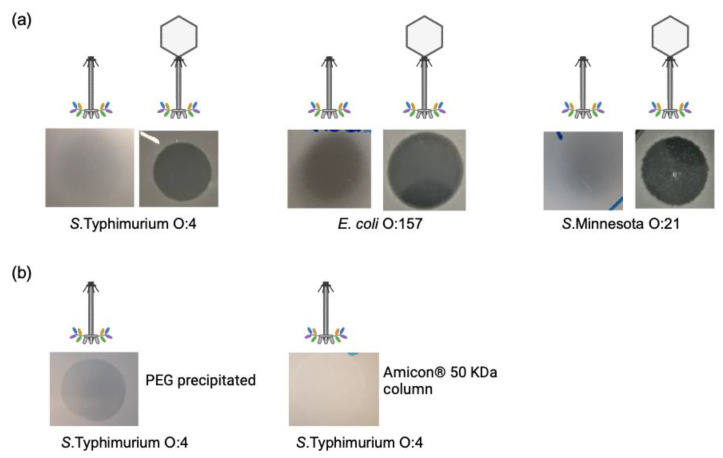
The effect of Tailocin on the native hosts of S117. (**a**) All three hosts of *S.* Typhimurium (strain LT2C (O:4)), *S.* Minnesota (strain JEO2 (O:21)), and *E. coli* (strain NTCT12900 (O:157)) were sensitive to Tailocin and the native phage S117. (**b**) The Tailocin effect on *S.* Typhimurium after a concentration of 1000 times using PEG and the Amicon^®^ 50 KDa column. A killing halo is visible, and there were no single plaques, confirming the presence of Tailocin particles.

To produce Tailocin particles, it is important to note that, once the Tailocins are produced, they must be harvested as soon as possible to avoid binding to a new host or cell debris and be irrecoverable. Therefore, we used MOI 1 as the best compromise to produce the highest amount of Tailocin particles at 40 min post-infection by infecting one liter culture of *S.* Typhimurium LT2C by S117Δ*portal*. The Tailocin particles produced were tested by spot assay onto the native hosts of phage S117 along with the native phage [24]. The harvested Tailocin particles produced a dim killing halo on all native S117 hosts and no clear lysis, as when phage S117 was spotted (Figure 5a). Furthermore, no single plaques were observed, confirming the production of Tailocin particles and no phage particles. Tailocin particles were then concentrated using PEG [38], and a dim killing halo and no single plaques were visible on a lawn of *S.* Typhimurium (Figure 5b). As the killing halo observed could have been the result of the presence of endolysin, Tailocin particles were also concentrated using column-size exclusion (50 KDa). Considering that S117 endolysin is 28 KDa (ORF79, Protein ID: AXC40792.1), it would not have been retained. Tailocin concentrated by column produced similar results as with PEG (Figure 5b), indicating that the killing halo is the result of Tailocin action. The halo produced by our PEG-concentrated Tailocins is comparable to our 10^−5^-dilution spot assay of the S117Δ*portal,* corresponding to 10^6^ pfu. Therefore, we can estimate that the amount of Tailocin that PEG concentrated is 10^6^ particles. Moreover, the concentration of Tailocin was further determined by mixing a successive dilution of 10^8^ up to 10^1^ cfu of *S.* Typhimurium LT2C and by counting the survival population. The estimated amount of Tailocin produced from one liter of culture and concentrated to 1 mL by PEG was also 10^6^ particles per mL, considering that one Tailocin kills one bacterial cell, as previously described for pyocins [10].

Visualization of the Tailocin particles was not attempted by TEM, as a minimum of 10^9^ particles of phage are usually required [39]. However, when serial dilutions of the concentrated Tailocin particles were spotted on a lawn of LT2C, we noticed a few single plaques, suggesting the presence of phage particles at 10^2^ pfu per mL in the Tailocin preparation. Surprisingly, plaque PCR showed that these phage particles contained the pECgRNA plasmid carrying the *cportal* gene used for complementation. The plaques were the result of a double infection by a transducing particle containing the *cportal* (pEcgRNA) and phage S117Δ*portal*, which allowed production of S117Δ*portal* particles at a low frequency. We tested both our stock of S117Δ*portal* and S117 and found that both were able to transduce full circular plasmids (pEcCas, pECgRNA) and chromosomal fragments from their host. We estimated the presence of 10^4^ transductants per plaque-forming unit, in agreement with previous work [40]. We then attempted to complement the *portal* on the chromosome to minimize the number of transducing particles containing the *cportal*, yet with little improvement. Thus, we reported the ability of this phage to transduce DNA, as previously described for other *Ackermannviridae* phages [40]. Overall, we successfully produced Tailocin particles from phage S117. 

### 3.5. Tailocin Particles Produced by Engineering Temperate Phages of S. Typhimurium

Temperate phages may be easier to engineer, as they are integrated in the host chromosomal genome. Seemingly, as for virulent phage S117, the deletion of structural head genes in all temperate phages would allow for the production of a cocktail of different Tailocins at once. Temperate phages Fels-1, -2 and Gifsy-1, -2 are *Salmonella*-temperate phages showing *Siphoviridae* and *Myoviridae* morphologies that could be transformed into Tailocins. 

#### 3.5.1. Bioinformatic Identification of Fels-1 and Fels-2, Putative Receptors

While temperate phage Gifsy-1 and -2 attach to OmpC as receptors [26], Fels-1 and -2 receptors were not identified. Considering that the tail fiber protein of Fels-1 (STM0926 in LT2 genome NC_003197) is 93% and 92%, identical to Gifsy-1 and Gifsy-2, respectively, we can assume that Fels-1 uses OmpC as a receptor. In contrast, Fels-2 encodes a tail fiber protein (STM2706 in LT2 genome NC_003197) with a C-terminal region similar to temperate phages SJ46 and ST64B, showing 84% and 72% identity, respectively. Since the tail fiber C-terminal part is responsible for binding to the bacterial receptor [41], we can assume Fels-2 would use the same receptor as phages SJ46 and ST64B. Unfortunately, neither SJ46 nor ST64B had their bacterial receptor characterized. Yet, as Fels-2 tail fiber is different from Fels-1 and Gifsy-1 and -2, we can assume that Fels-2 uses a different receptor. Genetically engineering these prophages into Tailocins would therefore transform *Salmonella* into a Tailocin production factory, producing a cocktail of contractile and non-contractile Tailocin particles, targeting the same host but likely different receptors.

#### 3.5.2. Genetic Engineering of Temperate Phages Fels-1, Fels-2, Gifsy-1, and Gifsy-2

**Figure 6:**
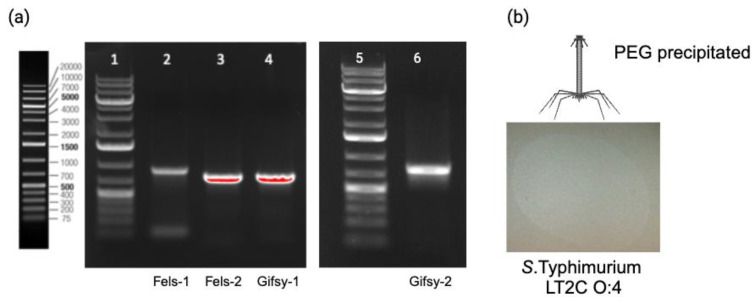
The construction and characterization of Tailocins originating from temperate phages Fels-1, Fels-2, Gifsy-1, and Gifsy-2. (**a**) The deletion of the *major capsid* genes from Fels-1, -2 and Gifsy-1, -2 in *S. enterica* 3674. Wells (1) and (5) Ladder 1Kb+, (2) Fels-1, (3) Fels-2, (4) Gifsy-1, (6) Gifsy-2. An amplification can only be the result of the integration of the HR fragment into the chromosome, as the forward primer binds to the chromosome outside the HR, and the reverse primer binds to the HR fragment inside the resistance gene inserted. All temperate phages were deleted for their *major capsid*. (**b**) The effect of Tailocin particles produced from temperate phages Fels-1, -2 and Gifsy-1, -2 was deleted for the *major capsid* gene on *S.* Typhimurium strain LT2C. A dim killing halo was visible, and there were no single plaques, confirming the presence of Tailocin particles produced from the engineered temperate phages of *S.* Typhimurium 3674.

To transform phages Fels-1, Fels-2, Gifsy-1, and Gifsy-2 into Tailocins, we successfully deleted their *major capsid* genes in *S.* Typhimurium 3674 by providing a PCR fragment containing a homologous recombination template deleted for the *capsid* gene (Figures S2–S5). The deletion of the *major capsid* genes was confirmed by PCR (Figure 6a). We then induced the production of Tailocin particles upon the activation of the temperate phages through mitomycin C treatment and concentrated it using PEG, as previously described for the S117Δ*portal*. We tested for the presence of Tailocin particles by spot assay on a lawn of *S.* Typhimurium strain LT2C and 3674. The presence of a dim killing halo was observed only on a lawn of LT2C (Figure 6b) but not on 3674. As *S.* Typhimurium LT2C is devoid of prophages, but not strain 3674, this may indicate a mechanism of superinfection exclusion. The amount of Tailocin particles produced was comparable to the amount obtained by engineering phage S117 (10^6^ particles per mL). It is probable that the low quantity of Tailocin particles obtained is in relation to the temperate phages’ RBPs targeting their own producer host. As previously observed for S117 Tailocin particles, due to the low quantity of particles obtained from temperate phages, we could not visualize the Tailocin by TEM pictures. In summary, we successfully produced Tailocin particles from temperate phage present in *S.* Typhimurium. 

## 4. Discussion

Phages have been used to successfully treat bacterial infection for a century in Eastern countries (e.g., Georgia [3]). Today, phages are starting to be accepted as viable complements/alternatives to antibiotic treatment in Western countries too, especially to treat antibiotic-resistant bacterial infection [42]. Yet, due to their replicative nature, phages are difficult to validate pharmacokinetically, as well as to set up administration schemes, and they are used mainly for compassionate treatments [42]. Interestingly, bacteria can also produce phage-like particles that they use to kill competing species [43]. These are called Tailocins and resemble head-less phage particles that kill by puncturing the bacteria membrane, resulting in death by dissipation of the proton-motive force and cellular collapse [44,45,46]. Being inspired by the natural Tailocins, we proposed that phages may be transformed into such bacterial-killing agents by preventing head formation or the head-to-tail connection of phage assembly. In this study, we genetically engineered virulent and temperate phages into Tailocins by deleting the *portal* or the *major capsid* genes using CRISPR-Cas9 and homologous recombination, respectively. 

Our engineering approach opens an avenue for transforming any type of phage into Tailocins. As a supplement, other approaches to producing Tailocin from phage particles may be considered. We previously attempted to produce Tailocin from phage S117 by osmotic shock and chemical treatment, without success [47], yet other phages may be transformed into Tailocins by these methods. Other genetic strategies to producing Tailocin exist, such as the use of RNA inhibition [48]. An antisense RNA produced constitutively within the host, prior to infection by the native phage, could prevent the head synthesis by complementary binding. We attempted this approach by targeting the *major capsid* gene of S117, without success. In addition, CRISPR-Cas9 can also be used to prevent RNA synthesis through CRISPR-Cas9 inhibition (CRISPRi) [49], where Cas9 is engineered to be deficient for its catalytic site (deadCas9), preventing its endonuclease activity, yet preserving its binding ability [50]. By targeting the promoter of the *portal* or the *major capsid* gene with dCas9, RNA polymerase may be prevented from producing the mRNAs of these genes. For phage S117, this approach was not successful, most likely due to few possibilities for guide design in the promoter region. In addition, the high level of DNA modification of the phage genome may prevent dCas9 from binding efficiently to the guide, similar to our many guides designed targeting the *portal* gene. Thus, these approaches may not be suitable for phages carrying modified DNA and deleting the *portal* or the *major capsid* genes using CRISPR-Cas9 and homologous recombination may be a more feasible way forward. 

For therapeutic applications, the concentration of Tailocin particles is of major importance. Using our experimental setup, a production of up to 10^6^ particles per mL, after a concentration step, was achieved for both engineered virulent and temperate phages. Still, the quantity of particles produced in our study was lower than what was obtained for Pyocin production [51]. This native Tailocin is encoded by the chromosome of *Pseudomonas*, and its production is thus comparable to Tailocins derived from temperate phages. However, previous work reported that Pyocin particles may bind and/or contract upon encountering cellular debris [16]. Thus, for improving production, we proposed changing the RBP of the engineered temperate phage to prevent Tailocin from binding to the producer host. This may result in upscaling the concentration of Tailocin particles up to a similar level as for Pyocin, where 10^11^ particles per mL of lysate can be obtained [16]. This may be sufficient for therapeutical application, as the recommended phage density is 10^8^ per mL [52]. Yet, considering the replicative nature of phages, it is expected that the concentration of Tailocin particles needed is higher. Indeed, it has been reported that 3.10^11^ particles of Pyocin R2 injected intra peritoneal rescued a mouse from a *Pseudomonas aeruginosa* infection [51]. This stresses the importance of a substantial number of Tailocin particles for therapeutic applications. Furthermore, the stability and purity of the Tailocins need to be assessed to ensure that preparations could be used for therapeutical purposes. This can be performed using dynamic light scattering (DLS), which was recently successfully employed to characterize the stability of phage preparation bioactivity [53]. 

Considering the huge number of phages present in the environment, we expect that many diverse phages can be transformed into a Tailocin to target any pathogenic or unwanted bacteria. For selecting phages to transform into Tailocins, both target bacterium, morphology of the phage, as well as the level of identified biological functions may be important. In our case, the *Akermannviridae* phage S117 was selected due to its contractile tail, characterized genome, and multiple TSPs targeting diverse bacteria, including the food-borne pathogen *Salmonella* [24]. Importantly, we successfully transformed this phage into a functional Tailocin by constructing the engineered phage S117Δ*portal*. Yet, after concentrating the Tailocin, a low number of transducing particles was observed in the preparation. Since transducing particles can transfer genetic material horizontally, such particles are undesirable in products for therapeutic applications. To overcome this, non-transducing phages could be used as a backbone for engineering. Alternatively, switching Tailocin production to a cell-free expression system may be more appropriate. We tried to produce full phage particles and Tailocins from phage S117 and our genetically engineered S117Δ*portal* in a cell-free system without success; yet the genome of phage S117 is quite large. However, cell-free expression systems have previously been used successfully to produce a therapeutic amount of phage particles [54,55]; still, our work highlights the need for the optimization of larger genomes, such as phage S117, as well-engineered DNA [56]. Overall, cell-free extract is an attractive strategy, as it relies only on the genetic material provided. Thus, when further optimized, it could be envisioned to produce Tailocin particles out of any phages deleted for their *portal* or *major capsid* gene. 

In summary, by optimizing engineering strategies as well as production, Tailocins represent an immense untapped reservoir of alternative antimicrobials that could be used in the fight against antibiotic-resistant bacteria.

## 5. Conclusions

With the increase of antibiotic-resistant infections worldwide, the need for new alternatives has never been more important. Tailocins are promising antimicrobials that have already been tested in vivo and proven successful. Our study showed that it is possible to engineer Tailocins from phages, both virulent and temperate. We expect this work to provide a starting point for extending the range of Tailocin applications and new antimicrobials to treat bacterial infection in the future. 

## Figures and Tables

**Table 1:** Predicted and tested efficiency of CRISPR-Cas9 guides targeting the S117 portal.

Name	Sequence	PAM	Strand	Position	On Target ^1^	Off Target ^2^	EOP LOG Reduction
gPortal1	CTTCCTGTGTAATCTCCGCG	AGG	−	1205	96	100	0
gPortal2	GGTTCTATAATCCTTCAGGG	CGG	+	168	87.8	100	0
gPortal3	ATAGAATGCGCGTTTCTCAG	GGG	−	865	70.7	100	0
gPortal4	CAATGATGTTGTCACCTGTG	AGG	+	311	74	100	0
gPortal5	GCATTCTGACCTTCGCGGCG	CGG	−	1036	62	100	0
gPortal6	TACCAGATTGGCCAGTGAAT	TGG	−	654	34	100	0
gPortal7	ATGGGGTTCTTCGGTTTGTT	TGG	+	36	0	100	0
gPortal8	CAACAATTCATCTTGTTTTT	GGG	−	85	19	100	0
gPortal9	TCTCAGCGTTAAAACAGTTG	TGG	−	230	32	100	0
gPortal10	CATAACTTCTTTGAAGCATT	CGG	−	388	38	100	0
gPortal11	ATCCCACGAATAAAAAAGGC	GGG	+	514	18	100	5
gPortal12	GAGAAGGCGATGCGTGAAGG	CGG	+	591	80	100	0
gPortal13	GGTGAAGAGCCATTGGCCAA	TGG	+	768	53	100	0
gPortal14	GAATACATGACCATGATGAT	GGG	+	942	38	100	2
gPortal15	GTGAATTCAAACTTTATAAA	CGG	-	1356	38	100	0
gPortal16	TTGGCCAATGGTATTGTCCC	AGG	+	760	45	100	1
gPortal17	CAACGCATATGACCGCACCAC	TGG	+	960	64	100	0
gPortal18	AAGAGCCGCCTCCAAGAGGA	AGG	+	1156	62	100	0
gPortal19	GTTAATTTTGAAAGGCGTTA	CGG	+	1302	44	100	2
gPortal20	AAGGTTCAAGCAGATGAAAC	TGG	+	1576	51	100	0

^1^ Spacer theoretical efficiency calculated using CrisprScan [36]. ^2^ Spacer specificity from using *S. enterica* ATCC14028.

## Data Availability

Data are contained within the article.

## Supplementary Materials

The following supporting information can be downloaded at: https://www.mdpi.com/article/10.3390/cells12222637/s1, Figure S1: pEcgRNA plasmid map to delete S117 portal gene; Figure S2: HR PCR fragment to delete Fels-1 in strain *S.* Typhimurium LT2C; Figure S3: HR PCR fragment to delete Fels-2 in strain *S.* Typhimurium LT2C; Figure S4: HR PCR fragment to delete Gifsy-1 in strain *S.* Typhimurium LT2C; Figure S5: HR PCR fragment to delete Gifsy-2 in strain *S.* Typhimurium LT2C. Table S1: Primers used in this study.
