# Supplementary figures and images for: Engineering of Salmonella Phages into Novel Antimicrobial Tailocins
Source: Cells. 2023 Nov 16;12(22):2637. doi: 10.3390/cells12222637 (PMC10670071; doi:10.3390/cells12222637)

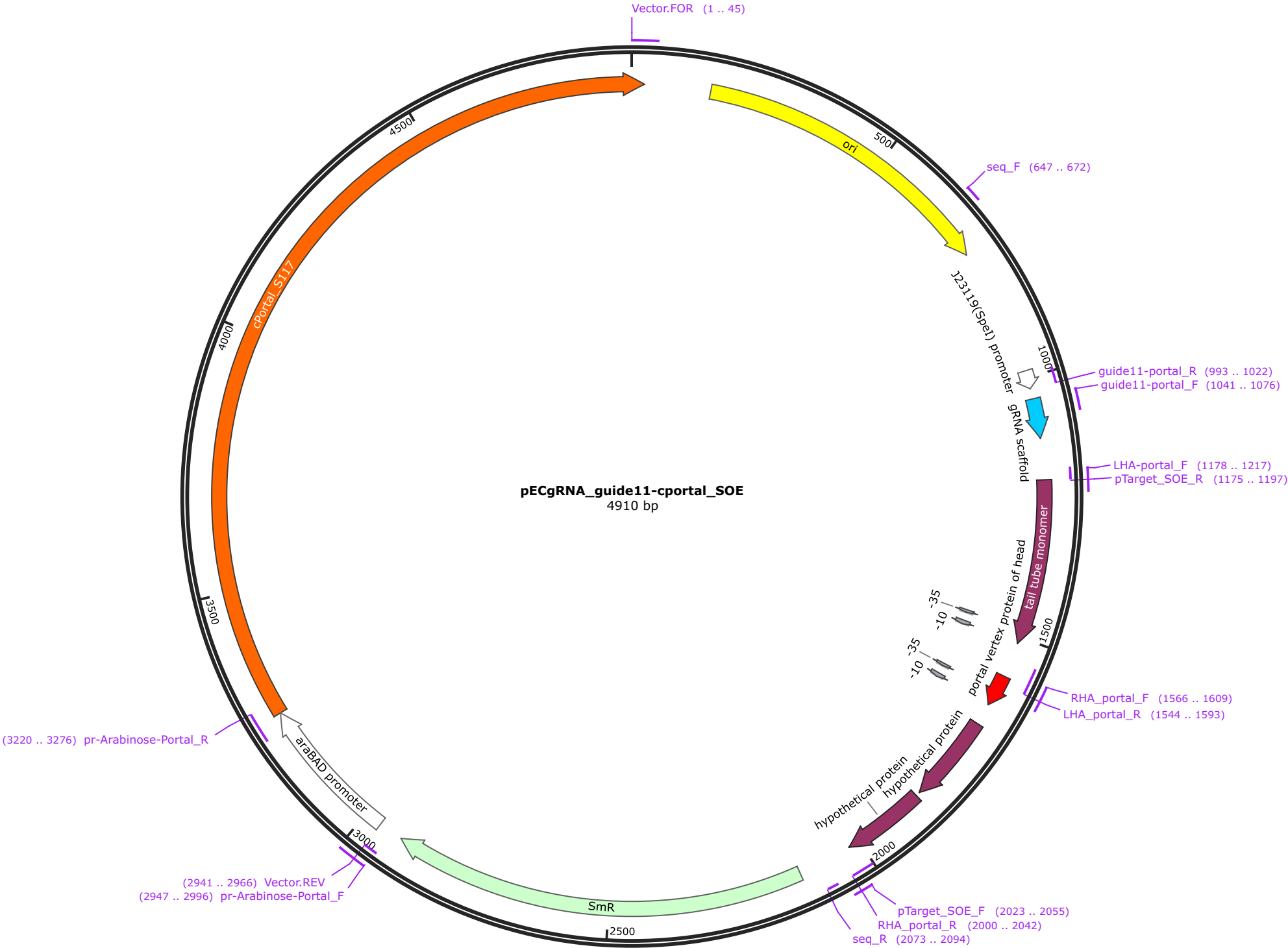

Supplement: Supplementary file 1 [file cells-12-02637-s001.zip › Figure S1.pdf]

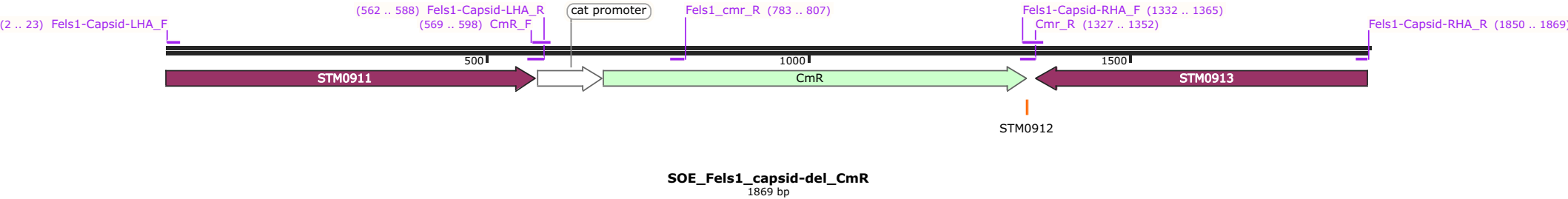

Supplement: Supplementary file 1 [file cells-12-02637-s001.zip › Figure S2.pdf]

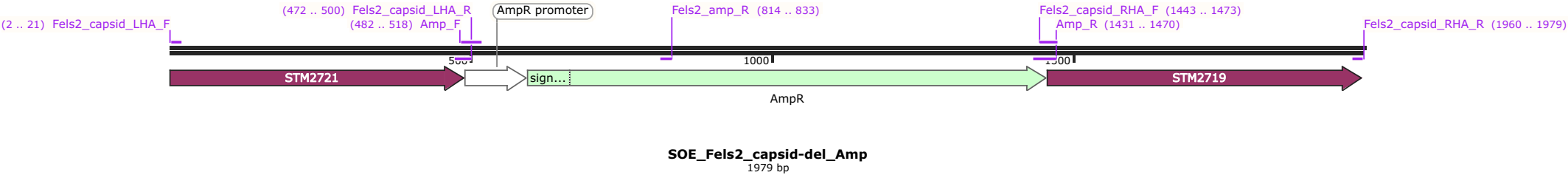

Supplement: Supplementary file 1 [file cells-12-02637-s001.zip › Figure S3.pdf]

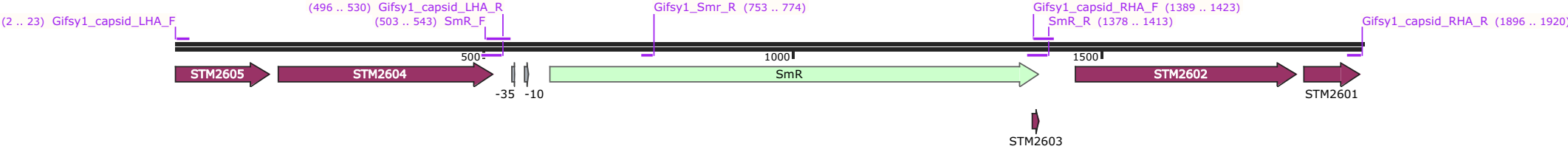

**SOE\_Gifsy1\_capsid-del\_SmR**  
1920 bp

Supplement: Supplementary file 1 [file cells-12-02637-s001.zip › Figure S4.pdf]

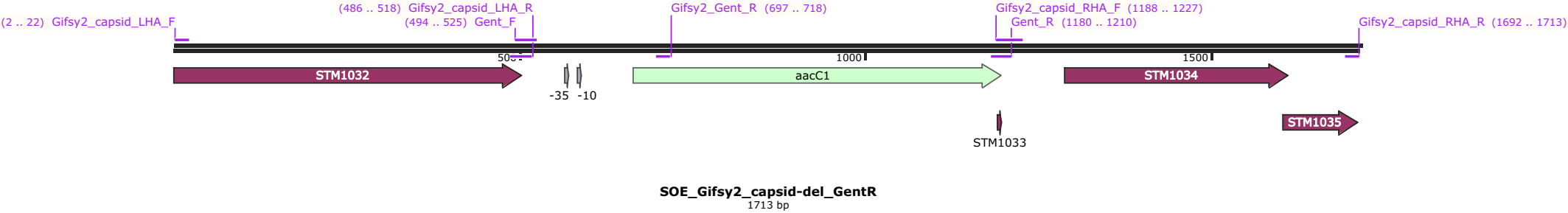

Supplement: Supplementary file 1 [file cells-12-02637-s001.zip › Figure S5.pdf]
